# Supplementary material for: LITOS: a versatile LED illumination tool for optogenetic stimulation
Source: Sci Rep. 2022 Jul 30;12:13139. doi: 10.1038/s41598-022-17312-x (PMC9338960; doi:10.1038/s41598-022-17312-x)
Supplement: Supplementary file 1 — Supplementary Information 1. [file 41598_2022_17312_MOESM1_ESM.html]

Cell based experiments to showcase LITOS


Code 

- Show All Code
- Hide All Code
- Download Rmd

# Cell based experiments to showcase LITOS

# Introduction

This is the data analysis code of the cell-based experiment (Figure 4 and 5) to showcase LITOS with the optogenetic stimulation of the MAPK/ERK pathway. The methodological details about the experiments and the description of the results can be found in the main text of the paper.

# Analyses of the experiments

## Loading packages and data


The meta-package `tidyverse` is used to prepare (e.g. with `dplyr`) and to plot (with `ggplot2`) the data.

The first step is to load the data.


```
data_western_blot <- read_csv("data_WesternBlot.csv")
data_dose_response <- read_csv("data_DoseResponse.csv")
data_dynamics <- read_csv("data_Dynamics.csv")
data_high_resolution <- read_csv("data_HighResolution.csv")
data_uniform_activation <- read_csv("data_Uniform.csv")
data_mini_screen <- read_csv("data_MiniScreen.csv")
```

## Uniform activation experiment

Plot the color-coded ERK activity (C/N - ratio) for each cell at its respective position in the well (Figure 4c).


```
data_plot <- data_uniform_activation %>%
  # Manually remove outliers
  filter(!(Image_Metadata_Well == "D09" & objNuc_ObjectNumber == 1) &
           !(Image_Metadata_Well == "D11" & objNuc_ObjectNumber == 207) & 
           !(Image_Metadata_Well == "D11" & objNuc_ObjectNumber == 2885)) %>% 
  
  # Rename columns
  mutate(X = objNuc_Location_Center_X, Y = objNuc_Location_Center_Y, 
         `C/N` = cytoVnuc.corr, 
         Timepoint = paste("t = ", Timepoint, "'", sep = ""), 
         Timepoint = factor(Timepoint, levels = c("t = 0'", "t = 3'", "t = 6'", "t = 9'", "t = 12'"))) %>% 
  
  # Limit the C/N to a min of 0.2 and a maximum of 1.5, so the color scale is more meaningful
  mutate(
    `C/N` = ifelse(`C/N` < 0.2, 0.2, `C/N`), 
    `C/N` = ifelse(`C/N` > 1.5, 1.5, `C/N`
  )) %>%
  ungroup()
          
# Plot the data^
ggplot(data_plot, aes(x = X, y = Y, col = `C/N`)) +
  geom_point(alpha = 0.85, shape=16, size =0.5) +
  scale_y_reverse() + 
  scale_color_gradient(high = "#f75555", low  = "#56B1F7") +
  theme_void() + theme(legend.position = "top") +
  facet_grid(. ~ Timepoint)
```

## Western blot experiment

Plot the color-coded ERK activity (C/N - ratio) for each timepoint so that they can be compared with the western blot results (Figure 4d).


```
data_plot <- data_western_blot %>% 
  group_by(RealTime) %>%
  summarise(meanResponse = mean(Response), medianResponse = median(Response))

ggplot(data_plot, aes(x = RealTime, y = 1, fill = meanResponse)) + 
  geom_tile() +
  scale_fill_gradient(high = "#f75555", low  = "#56B1F7") +
  theme(legend.position = "bottom") +
  xlab(xlab) + ylab(ylab)
```

## Dose-response experiment

Plot the dose response experiment (Figure 5a).


```
# Prepare data for plot
data_plot <- data_dose_response %>% 
  mutate(RealTime = factor(RealTime)) # change RealTime to a categorical variable

# Create data for blue square indicating stimulation
data_stim <- tibble(
  Cells = c("optoFGFR1", "optoFGFR1", "optoFGFR1", "optoFGFR1", "optoRAF", "optoRAF", "optoRAF", "optoRAF"),
  ExposureTime = c("0.5 min", "1 min", "2 min", "5 min", "0.5 min", "1 min", "2 min", "5 min"), 
  start = 0,
  end = c(0.5, 1, 2, 5, 0.5, 1, 2, 5),
  min = 0.2,
  max = 1.5
  ) 

# Plot 
ggplot(data_plot) +
  geom_boxplot(aes(x = RealTime, y = Response), outlier.shape = NA) +
  geom_rect(data=data_stim, aes(xmin = start+1, xmax = (end/3)+1, ymin = min, ymax = max), fill = "blue", alpha = 0.2) +
  geom_boxplot(aes(x = RealTime, y = Response), outlier.shape = NA) + # put boxplot over rectangles
  coord_cartesian(ylim = c(0.27, 1.3)) +
  facet_grid(Cells ~ ExposureTime) +
  theme_bw() + theme(legend.position = "bottom", text = element_text(family = "sans-serif", size = 8)) +
  xlab(xlab) + ylab(ylab)
```

## Mini drug screen

Plot the results of the mini drug screen which was performed with LITOS (Figure 5b).


```
# Calculate mean per time point
data_plot <- data_mini_screen %>%
  group_by(Drug, Concentration, RealTime) %>% 
  summarise(err = sd(Response)/sqrt(n()), Response = median(Response), .groups = "drop")

# Generate data to indicate stimulations
data_stim <- tibble(
  Drug = c("RAF709", "UO126", "SCH772984")
  ) 

ggplot(data_plot) +
  geom_rect(data = data_stim, aes(xmin = 0.3, xmax = 0.7, ymin = 0.2, ymax = 0.7), fill = "blue", alpha = 0.2) +
  geom_point(aes(x = RealTime, y = Response, col = Concentration)) +
  geom_line(aes(x = RealTime, y = Response, col = Concentration, group = paste(Concentration, Drug))) +
  coord_cartesian(ylim = c(0.44, 0.65)) +
  theme(text = element_text(family = "sans-serif", size = 8), legend.position = "top") +
  facet_wrap(~Drug) +
  xlab(xlab) + ylab(ylab)
```

## High temporal resolution experiment

Plot a single ERK activity pulse with high temporal resolution (Figure 5c).


```
data_plot <- data_high_resolution

# Generate data to indicate stimulations
dp_stim <- tibble(
  Drug = c("RAF709")
  ) 

ggplot(data_plot) + 
  geom_boxplot(aes(x = factor(Timepoint), y = cytoVnuc.corr), outlier.shape = NA) + 
  geom_rect(data=dp_stim, aes(xmin = 1.3, xmax = 1.5, ymin = 0, ymax = 1.6), fill = "blue", alpha = 0.2) +
  geom_boxplot(aes(x = factor(Timepoint), y = cytoVnuc.corr), outlier.shape = NA) +  # put boxplot over rectangles
  coord_cartesian(ylim = c(0.15, 1.438)) +
  theme(text = element_text(family = "sans-serif", size = 8)) +
  xlab(xlab) + ylab(ylab)
```

## Induction of temporal dynamics

Plot the dynamics which was generated with LITOS (Figure 5d).


```
# Calculate mean and SEM per time point
data_plot <- data_dynamics %>% 
  # mutate(RealTime = as.numeric(as.character(RealTime))) %>%
  group_by(RealTime, PulseInterval) %>%
  summarise(mean = mean(Response), sd = sd(Response), sem = sd(Response)/sqrt(n()), .groups = "drop")

# Generate data to indicate stimulations
min = min(data_plot$mean)
max = max(data_plot$mean)

data_stim <- tibble(
  PulseInterval = c("sustained", "every 20 min", "every 20 min", "every 20 min", "every 20 min"),
  start = c(4, 4, 24, 44 , 64),
  end = c(84, 4.4, 24.4, 44.4, 64.4),
  min = min-0.1,
  max = max+0.1
  ) 

# Plot 
ggplot(data_plot) +
  geom_rect(data=data_stim, aes(xmin = start, xmax = end, ymin = min, ymax = max), fill = "blue", alpha = 0.2) +
  geom_line(aes(x = RealTime, y = mean, group = PulseInterval)) +
  geom_errorbar(aes(x = RealTime, y = mean, ymin = mean - sem, ymax = mean + sem)) +
  facet_grid(PulseInterval ~ .) +
  theme(text = element_text(family = "sans-serif", size = 8)) +
  xlab(xlab) + ylab(ylab)
```

LS0tCnRpdGxlOiAiQ2VsbCBiYXNlZCBleHBlcmltZW50cyB0byBzaG93Y2FzZSBMSVRPUyIKb3V0cHV0OiBodG1sX25vdGVib29rCi0tLQoKIyBJbnRyb2R1Y3Rpb24KVGhpcyBpcyB0aGUgZGF0YSBhbmFseXNpcyBjb2RlIG9mIHRoZSBjZWxsLWJhc2VkIGV4cGVyaW1lbnQgKEZpZ3VyZSA0IGFuZCA1KSB0byBzaG93Y2FzZSBMSVRPUyB3aXRoIHRoZSBvcHRvZ2VuZXRpYyBzdGltdWxhdGlvbiBvZiB0aGUgTUFQSy9FUksgcGF0aHdheS4gVGhlIG1ldGhvZG9sb2dpY2FsIGRldGFpbHMgYWJvdXQgdGhlIGV4cGVyaW1lbnRzIGFuZCB0aGUgZGVzY3JpcHRpb24gb2YgdGhlIHJlc3VsdHMgY2FuIGJlIGZvdW5kIGluIHRoZSBtYWluIHRleHQgb2YgdGhlIHBhcGVyLiAKCiMgQW5hbHlzZXMgb2YgdGhlIGV4cGVyaW1lbnRzCiMjIExvYWRpbmcgcGFja2FnZXMgYW5kIGRhdGEKYGBge3IgU2V0IHVwIHZhcmlhYmxlcyAvIGxvYWQgZnVuY3Rpb24sIGVjaG89RkFMU0UsIG1lc3NhZ2U9RkFMU0V9CmxpYnJhcnkodGlkeXZlcnNlKQoKIyBTZXR0aW5ncyBmb3IgcGxvdHMKdGhlbWVfc2V0KHRoZW1lX2J3KCkpCnNjYWxlX2ZhY3RvciA9IDEuNQpuLnBsb3QuaGVpZ2h0ID0gNDAKbi5wbG90LndpZHRoID0gNjAKeGxhYiA9ICJUaW1lIFttaW5dIgp5bGFiID0gIkVSSy1LVFIgW0MvTl0iCgpgYGAKClRoZSBtZXRhLXBhY2thZ2UgYHRpZHl2ZXJzZWAgaXMgdXNlZCB0byBwcmVwYXJlIChlLmcuIHdpdGggYGRwbHlyYCkgYW5kIHRvIHBsb3QgKHdpdGggYGdncGxvdDJgKSB0aGUgZGF0YS4gCgpUaGUgZmlyc3Qgc3RlcCBpcyB0byBsb2FkIHRoZSBkYXRhLiAKYGBge3IsIG1lc3NhZ2U9RkFMU0V9CgpkYXRhX3dlc3Rlcm5fYmxvdCA8LSByZWFkX2NzdigiZGF0YV9XZXN0ZXJuQmxvdC5jc3YiKQpkYXRhX2Rvc2VfcmVzcG9uc2UgPC0gcmVhZF9jc3YoImRhdGFfRG9zZVJlc3BvbnNlLmNzdiIpCmRhdGFfZHluYW1pY3MgPC0gcmVhZF9jc3YoImRhdGFfRHluYW1pY3MuY3N2IikKZGF0YV9oaWdoX3Jlc29sdXRpb24gPC0gcmVhZF9jc3YoImRhdGFfSGlnaFJlc29sdXRpb24uY3N2IikKZGF0YV91bmlmb3JtX2FjdGl2YXRpb24gPC0gcmVhZF9jc3YoImRhdGFfVW5pZm9ybS5jc3YiKQpkYXRhX21pbmlfc2NyZWVuIDwtIHJlYWRfY3N2KCJkYXRhX01pbmlTY3JlZW4uY3N2IikKCmBgYAoKIyMgVW5pZm9ybSBhY3RpdmF0aW9uIGV4cGVyaW1lbnQKUGxvdCB0aGUgY29sb3ItY29kZWQgRVJLIGFjdGl2aXR5IChDL04gLSByYXRpbykgZm9yIGVhY2ggY2VsbCBhdCBpdHMgcmVzcGVjdGl2ZSBwb3NpdGlvbiBpbiB0aGUgd2VsbCAoRmlndXJlIDRjKS4gCgpgYGB7ciBjZWxscyBwb3N9CgpkYXRhX3Bsb3QgPC0gZGF0YV91bmlmb3JtX2FjdGl2YXRpb24gJT4lCiAgIyBNYW51YWxseSByZW1vdmUgb3V0bGllcnMKICBmaWx0ZXIoIShJbWFnZV9NZXRhZGF0YV9XZWxsID09ICJEMDkiICYgb2JqTnVjX09iamVjdE51bWJlciA9PSAxKSAmCiAgICAgICAgICAgIShJbWFnZV9NZXRhZGF0YV9XZWxsID09ICJEMTEiICYgb2JqTnVjX09iamVjdE51bWJlciA9PSAyMDcpICYgCiAgICAgICAgICAgIShJbWFnZV9NZXRhZGF0YV9XZWxsID09ICJEMTEiICYgb2JqTnVjX09iamVjdE51bWJlciA9PSAyODg1KSkgJT4lIAogIAogICMgUmVuYW1lIGNvbHVtbnMKICBtdXRhdGUoWCA9IG9iak51Y19Mb2NhdGlvbl9DZW50ZXJfWCwgWSA9IG9iak51Y19Mb2NhdGlvbl9DZW50ZXJfWSwgCiAgICAgICAgIGBDL05gID0gY3l0b1ZudWMuY29yciwgCiAgICAgICAgIFRpbWVwb2ludCA9IHBhc3RlKCJ0ID0gIiwgVGltZXBvaW50LCAiJyIsIHNlcCA9ICIiKSwgCiAgICAgICAgIFRpbWVwb2ludCA9IGZhY3RvcihUaW1lcG9pbnQsIGxldmVscyA9IGMoInQgPSAwJyIsICJ0ID0gMyciLCAidCA9IDYnIiwgInQgPSA5JyIsICJ0ID0gMTInIikpKSAlPiUgCiAgCiAgIyBMaW1pdCB0aGUgQy9OIHRvIGEgbWluIG9mIDAuMiBhbmQgYSBtYXhpbXVtIG9mIDEuNSwgc28gdGhlIGNvbG9yIHNjYWxlIGlzIG1vcmUgbWVhbmluZ2Z1bAogIG11dGF0ZSgKICAgIGBDL05gID0gaWZlbHNlKGBDL05gIDwgMC4yLCAwLjIsIGBDL05gKSwgCiAgICBgQy9OYCA9IGlmZWxzZShgQy9OYCA+IDEuNSwgMS41LCBgQy9OYAogICkpICU+JQogIHVuZ3JvdXAoKQogICAgICAgICAgCiMgUGxvdCB0aGUgZGF0YV4KZ2dwbG90KGRhdGFfcGxvdCwgYWVzKHggPSBYLCB5ID0gWSwgY29sID0gYEMvTmApKSArCiAgZ2VvbV9wb2ludChhbHBoYSA9IDAuODUsIHNoYXBlPTE2LCBzaXplID0wLjUpICsKICBzY2FsZV95X3JldmVyc2UoKSArIAogIHNjYWxlX2NvbG9yX2dyYWRpZW50KGhpZ2ggPSAiI2Y3NTU1NSIsIGxvdyAgPSAiIzU2QjFGNyIpICsKICB0aGVtZV92b2lkKCkgKyB0aGVtZShsZWdlbmQucG9zaXRpb24gPSAidG9wIikgKwogIGZhY2V0X2dyaWQoLiB+IFRpbWVwb2ludCkKCmBgYAoKIyMgV2VzdGVybiBibG90IGV4cGVyaW1lbnQKUGxvdCB0aGUgY29sb3ItY29kZWQgRVJLIGFjdGl2aXR5IChDL04gLSByYXRpbykgZm9yIGVhY2ggdGltZXBvaW50IHNvIHRoYXQgdGhleSBjYW4gYmUgY29tcGFyZWQgd2l0aCB0aGUgd2VzdGVybiBibG90IHJlc3VsdHMgKEZpZ3VyZSA0ZCkuIAoKYGBge3IgVGltZXNlcmllcyB0b2dlaHRlciwgZXZhbCA9IEZBTFNFfQoKZGF0YV9wbG90IDwtIGRhdGFfd2VzdGVybl9ibG90ICU+JSAKICBncm91cF9ieShSZWFsVGltZSkgJT4lCiAgc3VtbWFyaXNlKG1lYW5SZXNwb25zZSA9IG1lYW4oUmVzcG9uc2UpLCBtZWRpYW5SZXNwb25zZSA9IG1lZGlhbihSZXNwb25zZSkpCgpnZ3Bsb3QoZGF0YV9wbG90LCBhZXMoeCA9IFJlYWxUaW1lLCB5ID0gMSwgZmlsbCA9IG1lYW5SZXNwb25zZSkpICsgCiAgZ2VvbV90aWxlKCkgKwogIHNjYWxlX2ZpbGxfZ3JhZGllbnQoaGlnaCA9ICIjZjc1NTU1IiwgbG93ICA9ICIjNTZCMUY3IikgKwogIHRoZW1lKGxlZ2VuZC5wb3NpdGlvbiA9ICJib3R0b20iKSArCiAgeGxhYih4bGFiKSArIHlsYWIoeWxhYikKCmBgYAoKCiMjIERvc2UtcmVzcG9uc2UgZXhwZXJpbWVudApQbG90IHRoZSBkb3NlIHJlc3BvbnNlIGV4cGVyaW1lbnQgKEZpZ3VyZSA1YSkuIAoKYGBge3IgRG9zZVJlc3BvbnNlfQoKIyBQcmVwYXJlIGRhdGEgZm9yIHBsb3QKZGF0YV9wbG90IDwtIGRhdGFfZG9zZV9yZXNwb25zZSAlPiUgCiAgbXV0YXRlKFJlYWxUaW1lID0gZmFjdG9yKFJlYWxUaW1lKSkgIyBjaGFuZ2UgUmVhbFRpbWUgdG8gYSBjYXRlZ29yaWNhbCB2YXJpYWJsZQoKIyBDcmVhdGUgZGF0YSBmb3IgYmx1ZSBzcXVhcmUgaW5kaWNhdGluZyBzdGltdWxhdGlvbgpkYXRhX3N0aW0gPC0gdGliYmxlKAogIENlbGxzID0gYygib3B0b0ZHRlIxIiwgIm9wdG9GR0ZSMSIsICJvcHRvRkdGUjEiLCAib3B0b0ZHRlIxIiwgIm9wdG9SQUYiLCAib3B0b1JBRiIsICJvcHRvUkFGIiwgIm9wdG9SQUYiKSwKICBFeHBvc3VyZVRpbWUgPSBjKCIwLjUgbWluIiwgIjEgbWluIiwgIjIgbWluIiwgIjUgbWluIiwgIjAuNSBtaW4iLCAiMSBtaW4iLCAiMiBtaW4iLCAiNSBtaW4iKSwgCiAgc3RhcnQgPSAwLAogIGVuZCA9IGMoMC41LCAxLCAyLCA1LCAwLjUsIDEsIDIsIDUpLAogIG1pbiA9IDAuMiwKICBtYXggPSAxLjUKICApIAoKIyBQbG90IApnZ3Bsb3QoZGF0YV9wbG90KSArCiAgZ2VvbV9ib3hwbG90KGFlcyh4ID0gUmVhbFRpbWUsIHkgPSBSZXNwb25zZSksIG91dGxpZXIuc2hhcGUgPSBOQSkgKwogIGdlb21fcmVjdChkYXRhPWRhdGFfc3RpbSwgYWVzKHhtaW4gPSBzdGFydCsxLCB4bWF4ID0gKGVuZC8zKSsxLCB5bWluID0gbWluLCB5bWF4ID0gbWF4KSwgZmlsbCA9ICJibHVlIiwgYWxwaGEgPSAwLjIpICsKICBnZW9tX2JveHBsb3QoYWVzKHggPSBSZWFsVGltZSwgeSA9IFJlc3BvbnNlKSwgb3V0bGllci5zaGFwZSA9IE5BKSArICMgcHV0IGJveHBsb3Qgb3ZlciByZWN0YW5nbGVzCiAgY29vcmRfY2FydGVzaWFuKHlsaW0gPSBjKDAuMjcsIDEuMykpICsKICBmYWNldF9ncmlkKENlbGxzIH4gRXhwb3N1cmVUaW1lKSArCiAgdGhlbWVfYncoKSArIHRoZW1lKGxlZ2VuZC5wb3NpdGlvbiA9ICJib3R0b20iLCB0ZXh0ID0gZWxlbWVudF90ZXh0KGZhbWlseSA9ICJzYW5zLXNlcmlmIiwgc2l6ZSA9IDgpKSArCiAgeGxhYih4bGFiKSArIHlsYWIoeWxhYikKCmBgYAojIyBNaW5pIGRydWcgc2NyZWVuClBsb3QgdGhlIHJlc3VsdHMgb2YgdGhlIG1pbmkgZHJ1ZyBzY3JlZW4gd2hpY2ggd2FzIHBlcmZvcm1lZCB3aXRoIExJVE9TIChGaWd1cmUgNWIpLgoKYGBge3IgTWluaVNjcmVlbn0KCiMgQ2FsY3VsYXRlIG1lYW4gcGVyIHRpbWUgcG9pbnQKZGF0YV9wbG90IDwtIGRhdGFfbWluaV9zY3JlZW4gJT4lCiAgZ3JvdXBfYnkoRHJ1ZywgQ29uY2VudHJhdGlvbiwgUmVhbFRpbWUpICU+JSAKICBzdW1tYXJpc2UoZXJyID0gc2QoUmVzcG9uc2UpL3NxcnQobigpKSwgUmVzcG9uc2UgPSBtZWRpYW4oUmVzcG9uc2UpLCAuZ3JvdXBzID0gImRyb3AiKQoKIyBHZW5lcmF0ZSBkYXRhIHRvIGluZGljYXRlIHN0aW11bGF0aW9ucwpkYXRhX3N0aW0gPC0gdGliYmxlKAogIERydWcgPSBjKCJSQUY3MDkiLCAiVU8xMjYiLCAiU0NINzcyOTg0IikKICApIAoKZ2dwbG90KGRhdGFfcGxvdCkgKwogIGdlb21fcmVjdChkYXRhID0gZGF0YV9zdGltLCBhZXMoeG1pbiA9IDAuMywgeG1heCA9IDAuNywgeW1pbiA9IDAuMiwgeW1heCA9IDAuNyksIGZpbGwgPSAiYmx1ZSIsIGFscGhhID0gMC4yKSArCiAgZ2VvbV9wb2ludChhZXMoeCA9IFJlYWxUaW1lLCB5ID0gUmVzcG9uc2UsIGNvbCA9IENvbmNlbnRyYXRpb24pKSArCiAgZ2VvbV9saW5lKGFlcyh4ID0gUmVhbFRpbWUsIHkgPSBSZXNwb25zZSwgY29sID0gQ29uY2VudHJhdGlvbiwgZ3JvdXAgPSBwYXN0ZShDb25jZW50cmF0aW9uLCBEcnVnKSkpICsKICBjb29yZF9jYXJ0ZXNpYW4oeWxpbSA9IGMoMC40NCwgMC42NSkpICsKICB0aGVtZSh0ZXh0ID0gZWxlbWVudF90ZXh0KGZhbWlseSA9ICJzYW5zLXNlcmlmIiwgc2l6ZSA9IDgpLCBsZWdlbmQucG9zaXRpb24gPSAidG9wIikgKwogIGZhY2V0X3dyYXAofkRydWcpICsKICB4bGFiKHhsYWIpICsgeWxhYih5bGFiKQoKYGBgCgoKCiMjIEhpZ2ggdGVtcG9yYWwgcmVzb2x1dGlvbiBleHBlcmltZW50ClBsb3QgYSBzaW5nbGUgRVJLIGFjdGl2aXR5IHB1bHNlIHdpdGggaGlnaCB0ZW1wb3JhbCByZXNvbHV0aW9uIChGaWd1cmUgNWMpLgoKYGBge3IgSGlnaCB0ZW1wb3JhbCBSZXNvbHV0aW9ufQoKZGF0YV9wbG90IDwtIGRhdGFfaGlnaF9yZXNvbHV0aW9uCgojIEdlbmVyYXRlIGRhdGEgdG8gaW5kaWNhdGUgc3RpbXVsYXRpb25zCmRwX3N0aW0gPC0gdGliYmxlKAogIERydWcgPSBjKCJSQUY3MDkiKQogICkgCgpnZ3Bsb3QoZGF0YV9wbG90KSArIAogIGdlb21fYm94cGxvdChhZXMoeCA9IGZhY3RvcihUaW1lcG9pbnQpLCB5ID0gY3l0b1ZudWMuY29yciksIG91dGxpZXIuc2hhcGUgPSBOQSkgKyAKICBnZW9tX3JlY3QoZGF0YT1kcF9zdGltLCBhZXMoeG1pbiA9IDEuMywgeG1heCA9IDEuNSwgeW1pbiA9IDAsIHltYXggPSAxLjYpLCBmaWxsID0gImJsdWUiLCBhbHBoYSA9IDAuMikgKwogIGdlb21fYm94cGxvdChhZXMoeCA9IGZhY3RvcihUaW1lcG9pbnQpLCB5ID0gY3l0b1ZudWMuY29yciksIG91dGxpZXIuc2hhcGUgPSBOQSkgKyAgIyBwdXQgYm94cGxvdCBvdmVyIHJlY3RhbmdsZXMKICBjb29yZF9jYXJ0ZXNpYW4oeWxpbSA9IGMoMC4xNSwgMS40MzgpKSArCiAgdGhlbWUodGV4dCA9IGVsZW1lbnRfdGV4dChmYW1pbHkgPSAic2Fucy1zZXJpZiIsIHNpemUgPSA4KSkgKwogIHhsYWIoeGxhYikgKyB5bGFiKHlsYWIpCgpgYGAKCiMjIEluZHVjdGlvbiBvZiB0ZW1wb3JhbCBkeW5hbWljcwpQbG90IHRoZSBkeW5hbWljcyB3aGljaCB3YXMgZ2VuZXJhdGVkIHdpdGggTElUT1MgKEZpZ3VyZSA1ZCkuCgpgYGB7ciBEYW55bWljc30KCiMgQ2FsY3VsYXRlIG1lYW4gYW5kIFNFTSBwZXIgdGltZSBwb2ludApkYXRhX3Bsb3QgPC0gZGF0YV9keW5hbWljcyAlPiUgCiAgIyBtdXRhdGUoUmVhbFRpbWUgPSBhcy5udW1lcmljKGFzLmNoYXJhY3RlcihSZWFsVGltZSkpKSAlPiUKICBncm91cF9ieShSZWFsVGltZSwgUHVsc2VJbnRlcnZhbCkgJT4lCiAgc3VtbWFyaXNlKG1lYW4gPSBtZWFuKFJlc3BvbnNlKSwgc2QgPSBzZChSZXNwb25zZSksIHNlbSA9IHNkKFJlc3BvbnNlKS9zcXJ0KG4oKSksIC5ncm91cHMgPSAiZHJvcCIpCgojIEdlbmVyYXRlIGRhdGEgdG8gaW5kaWNhdGUgc3RpbXVsYXRpb25zCm1pbiA9IG1pbihkYXRhX3Bsb3QkbWVhbikKbWF4ID0gbWF4KGRhdGFfcGxvdCRtZWFuKQoKZGF0YV9zdGltIDwtIHRpYmJsZSgKICBQdWxzZUludGVydmFsID0gYygic3VzdGFpbmVkIiwgImV2ZXJ5IDIwIG1pbiIsICJldmVyeSAyMCBtaW4iLCAiZXZlcnkgMjAgbWluIiwgImV2ZXJ5IDIwIG1pbiIpLAogIHN0YXJ0ID0gYyg0LCA0LCAyNCwgNDQgLCA2NCksCiAgZW5kID0gYyg4NCwgNC40LCAyNC40LCA0NC40LCA2NC40KSwKICBtaW4gPSBtaW4tMC4xLAogIG1heCA9IG1heCswLjEKICApIAoKIyBQbG90IApnZ3Bsb3QoZGF0YV9wbG90KSArCiAgZ2VvbV9yZWN0KGRhdGE9ZGF0YV9zdGltLCBhZXMoeG1pbiA9IHN0YXJ0LCB4bWF4ID0gZW5kLCB5bWluID0gbWluLCB5bWF4ID0gbWF4KSwgZmlsbCA9ICJibHVlIiwgYWxwaGEgPSAwLjIpICsKICBnZW9tX2xpbmUoYWVzKHggPSBSZWFsVGltZSwgeSA9IG1lYW4sIGdyb3VwID0gUHVsc2VJbnRlcnZhbCkpICsKICBnZW9tX2Vycm9yYmFyKGFlcyh4ID0gUmVhbFRpbWUsIHkgPSBtZWFuLCB5bWluID0gbWVhbiAtIHNlbSwgeW1heCA9IG1lYW4gKyBzZW0pKSArCiAgZmFjZXRfZ3JpZChQdWxzZUludGVydmFsIH4gLikgKwogIHRoZW1lKHRleHQgPSBlbGVtZW50X3RleHQoZmFtaWx5ID0gInNhbnMtc2VyaWYiLCBzaXplID0gOCkpICsKICB4bGFiKHhsYWIpICsgeWxhYih5bGFiKQoKYGBgCgoK
