# Supplementary material for: LITOS: a versatile LED illumination tool for optogenetic stimulation
Source: Sci Rep. 2022 Jul 30;12:13139. doi: 10.1038/s41598-022-17312-x (PMC9338960; doi:10.1038/s41598-022-17312-x)
Supplement: Supplementary file 11 — Supplementary Information 11. [file 41598_2022_17312_MOESM11_ESM.pdf]

LITOS - a versatile LED illumination tool for optogenetic stimulation

Thomas Christoph Höhener[1,+], Alex Erich Landolt[1,2,+], Coralie Dessauges[1], Lucien Hinderling[1], Paolo Armando Gagliardi[1], Olivier Pertz[1,*]

[1] Institute of Cell Biology, University of Bern, 3012 Bern, Switzerland
[2] Current address: ETH Zurich, Department of Biosystems Science and Engineering, 4058 Basel, Switzerland
[*] Corresponding author: olivier.pertz@unibe.ch
[+] these authors contributed equally to this work

Supplementary Figure S1

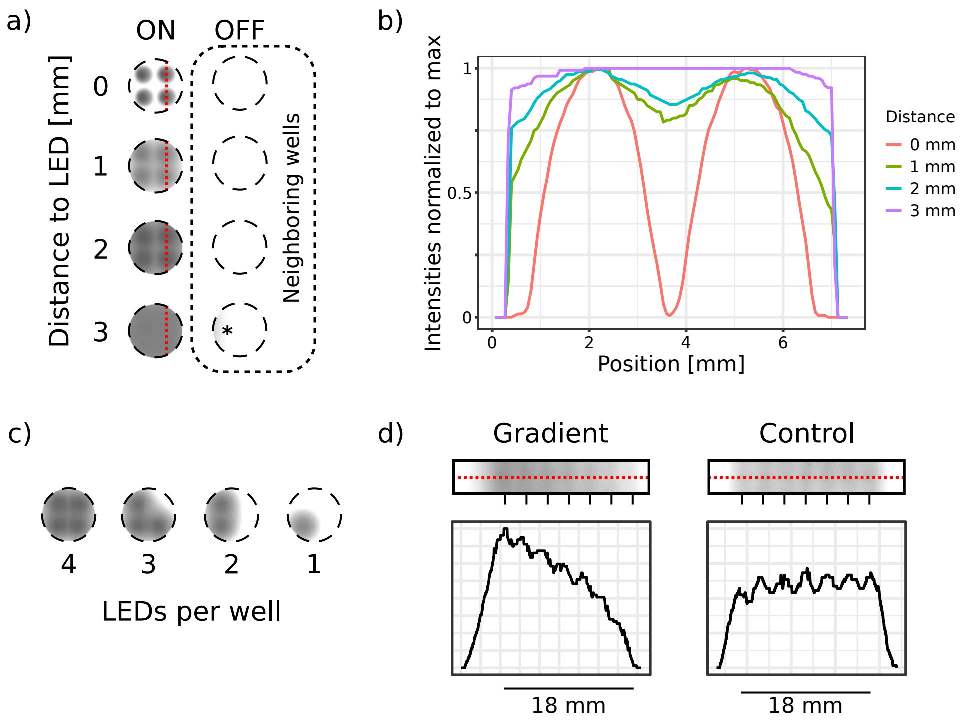

Figure S1: Measurement of the illumination generated by LITOS. a) Scan of photographic paper developed after being exposed to the light produced by 4 LEDs for 1 s at 23.5 % intensity (negative image of the illumination). The experiment was repeated with the photographic paper at different distances from the LEDs. The circles on the left represent the area of a well of a 96 well plate illuminated by the 4 LEDs. The circles on the right represent the illumination of a neighboring well. The asterisk highlights the illumination spillover in the neighboring well at 3 mm distance. b) Measurement of light intensity profiles along the dashed red line in "panel a". c) Scans of photographic paper exposed to 1 second of 23.5 % intensity blue light produced by a different number (1-4) of LEDs at 2 mm distance. d) Above: Cropped scans of photographic paper exposed to blue light produced by 7x7 LEDs at 2 mm distance for 1 s. On the left, the intensity of the LEDs was set to create a linear gradient (78.4 % to 19.6 % intensity), while on the right it was set to create uniform illumination (39.2 % intensity). The vertical lines indicate the position of the LEDs on the horizontal axis. Below: The charts represent intensity profiles along the dashed red line.

Supplementary Figure S2

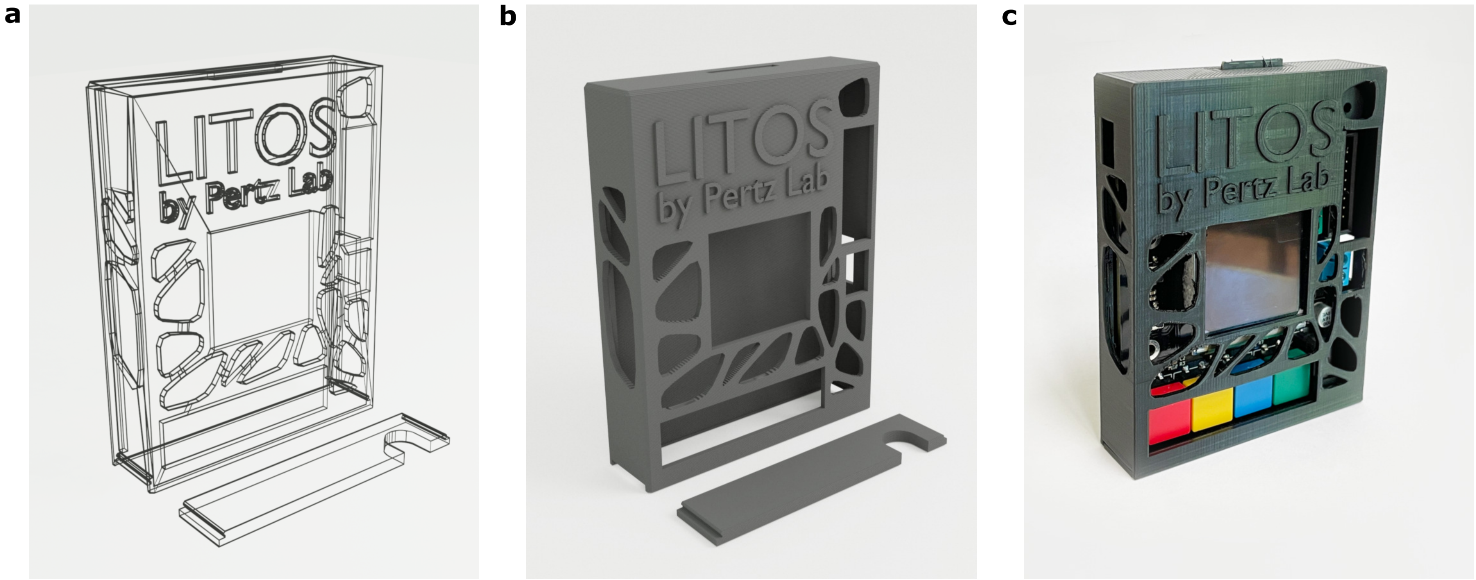

Figure S2: 3D printed case for the LITOS control unit. The design is easy to print and consist of two parts which can be assembled without tools or glue. The STL files are available on GitHub. a, b) Reduced wireframe model and 3D rendering of case, created in open source modelling and rendering package *Blender*. c) Photo of 3D printed case with inside the LITOS control unit.

Supplementary Figure S3

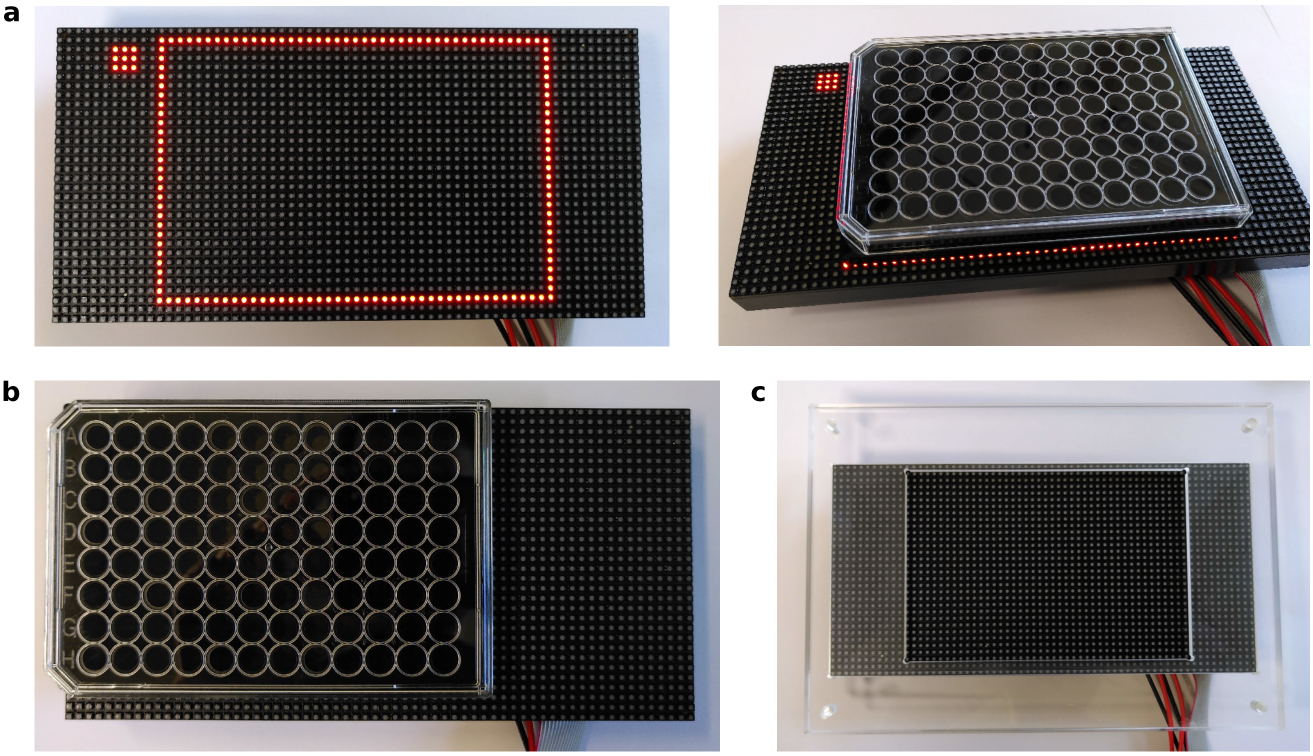

Figure S3: Different possibilities to align a well plate on the LED matrix. a) A way to align (especially in the dark) a multi-well plate on the LED matrix of LITOS is to use LITOS to show a reference outline on the matrix. The 3 by 3 illuminated square indicates the upper left-corner of the multi-well plate. b) Alignment of the plate to the top left corner of the LED matrix. c) A plexiglass mask is used to position one multi-well plate on the LED matrix (the plexiglass mask to position two multi-well plates is shown in figure 2c). The position of the illumination patterns can be set in the configuration interface according to the alignment approach.

Supplementary Figure S4

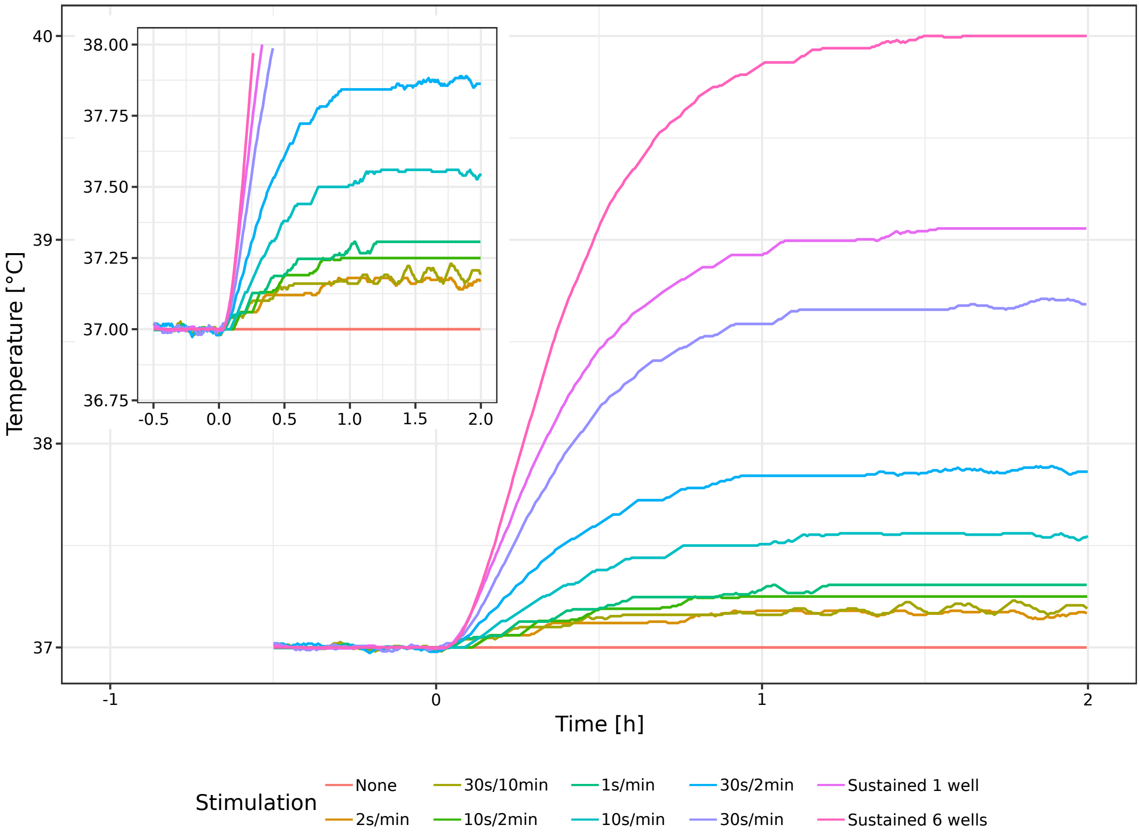

Figure S4: Temperature increase of cell culture medium due to different illumination frequencies. The temperature increase in an incubator due to different illumination intervals was determined by using an Arduino device equipped with multiple DS18B20 waterproof temperature sensors. The temperature sensors were placed in a well (6 well plate) filled with 3 ml water. The color of the line indicates the illumination interval, e.g. “10s/2min” means a 10 s pulse of blue light every 2 minutes.

Supplementary Figure S5

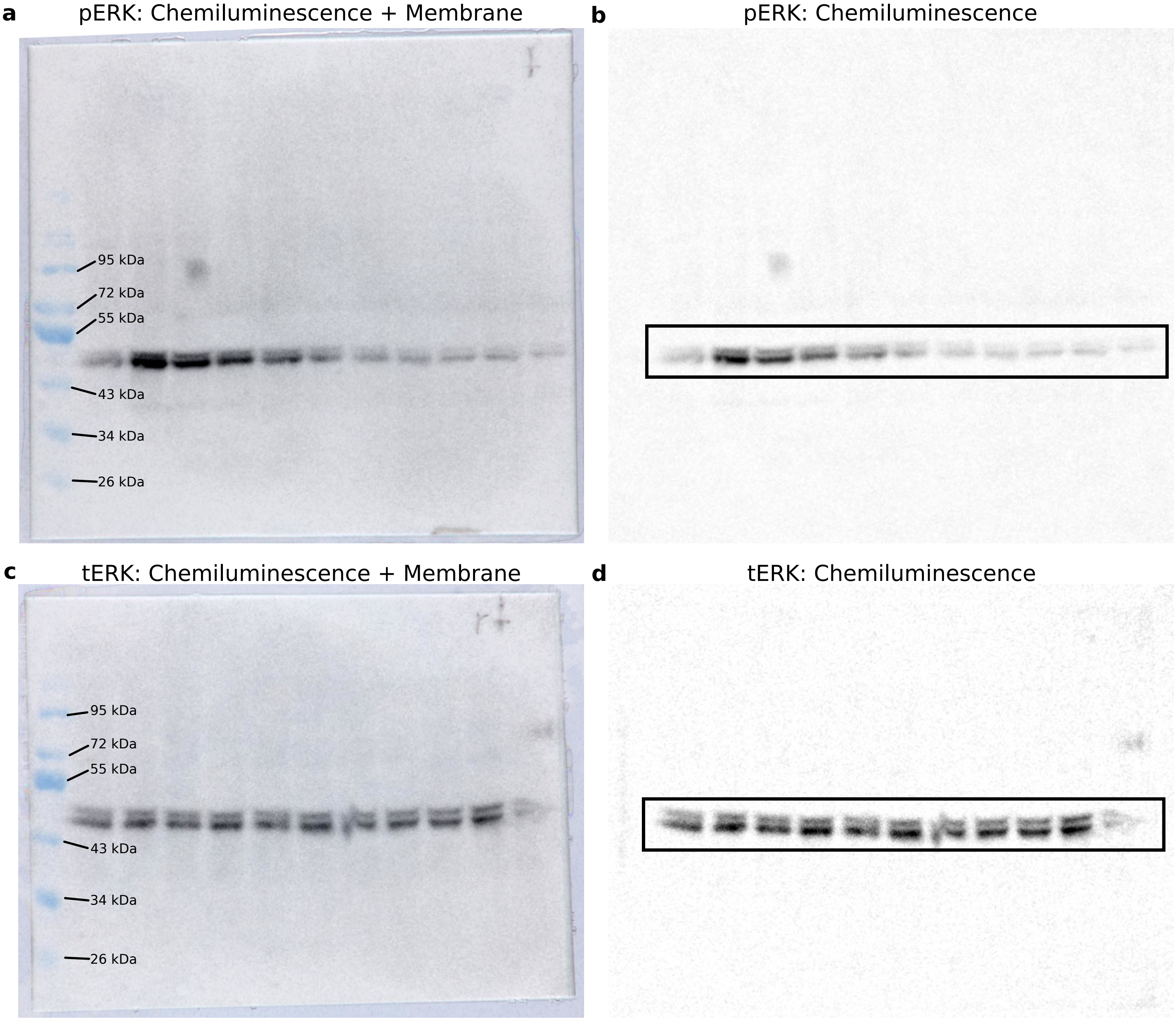

Figure S5: Uncropped plots from Figure 4d. Western blot against phosphorylated (a, b) and total ERK (c, d). Time series from 0 min to 27 min after optogenetic stimulation, with 3 min resolution.

Supplementary Figure S6

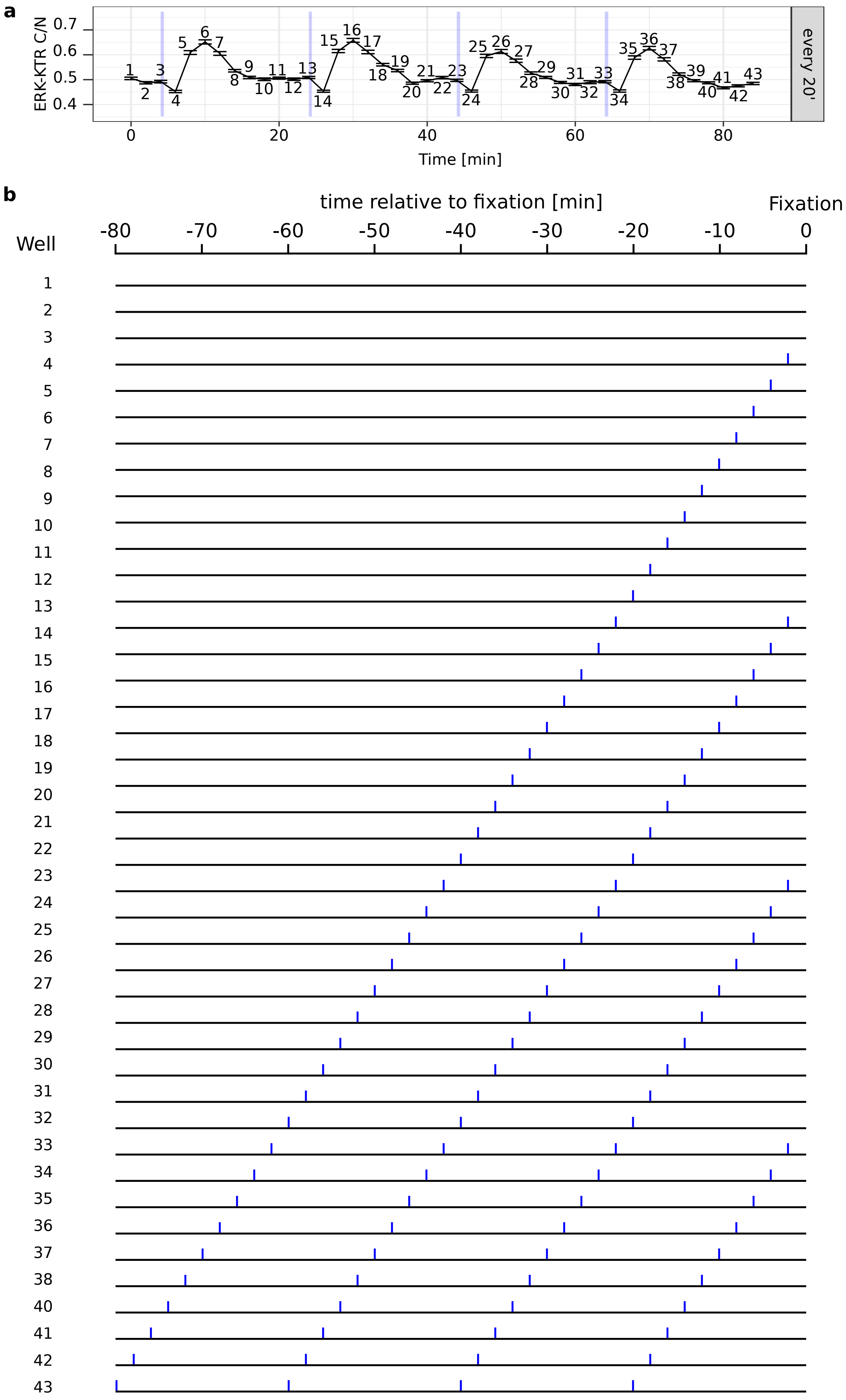

Figure S6: Visual representation of the illumination pattern of Figure 5d (low frequency). a) Pseudo time series of the low frequency stimulation from Figure 5d. For each time-point, the corresponding well is indicated with a number. b) Illumination pattern relative to the time of fixation (all at the same time/end of experiment) for each well that was used to obtain the pseudo time series (panel a). Blue lines represent a 10 s pulse with blue light.

Supplementary Figure S7

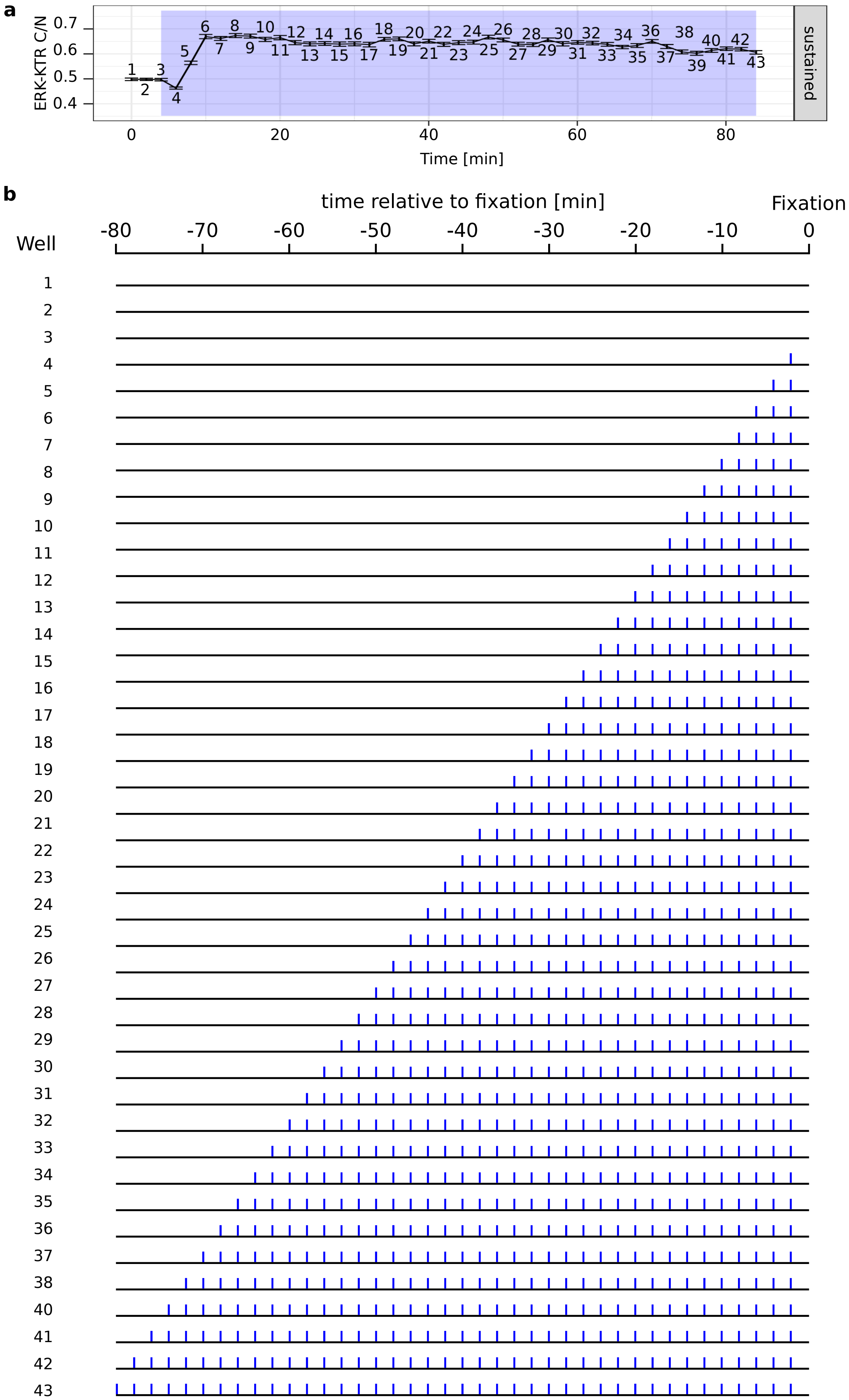

Figure S7: Visual representation of the illumination pattern of Figure 5d (high frequency). a) Pseudo time series of the high frequency stimulation from Figure 5d. For each time-point, the corresponding well is indicated with a number. b) Illumination pattern relative to the time of fixation (all at the same time/end of experiment) for each well that was used to obtain the pseudo time series (panel a). Blue lines represent a 10 s pulse with blue light.

Supplementary Table S1

Table S1: Comparison with other devices. Values of other devices according to published data.

Ref.	Power density	Intensity levels	Temporal resolution	Format	Heating
LITOS	135 µW/cm^2	256 levels	Software: 1 s<br>Hardware: 50 ms	6-96 wells<br>others	Normal use <0.3°C<br>Intense use <3°C
9	1000 µW/cm^2<br>2000 µW/cm2	0.0024 µW/cm^2<br>0.005 µW/cm^2	1-10 ms	24 wells	no effect measured
10	130 µW/cm^2	n/a	n/a	96 wells	n/a
11	n/a	4096 levels	1 ms	24 wells	n/a
12	4 mW/cm^2	4096 levels	n/a	96 wells	<4 °C
LUMOS	>1.9 mW/cm^2	0-100%	100 ms	96 wells	n/a

Supplementary Movie S1
Movie S1: Movie showing a stimulation pattern generated by LITOS for 96 well plates, in which every column is stimulated sequentially. Illumination with blue light at maximum intensity.
